# Health facility users’ knowledge, perceptions, and practices about infant feeding in the context of option B+ in South Africa: a qualitative study

**DOI:** 10.1186/s13006-022-00526-0

**Published:** 2022-12-20

**Authors:** Duduzile Faith Nsibande, Vuyolwethu Magasana, Wanga Zembe, Gurpreet Kindra, Mary Mogashoa, Ameena Goga, Vundli Ramokolo

**Affiliations:** 1 Health Systems Research Unit (HSRU), South African Medical Research Council (SAMRC), Cape Town, South Africa; 2 HIV and other Infectious Diseases Research Unit (HIDRU), SAMRC, Cape Town, South Africa; 3 United States Centers for Disease Control and Prevention, Pretoria, South Africa; 4 Department of Paediatrics and Child Health, University of Pretoria, Pretoria, South Africa

**Keywords:** Option B +, Infant feeding, Breastfeeding, Exclusive breastfeeding, Mixed feeding, Policy (6).

## Abstract

**Background:**

HIV and sub-optimal infant feeding practices remain important threats to child growth, development, and survival in low- and middle-income countries. To our knowledge, few studies have explored health service users’ perspective of infant feeding in the context of WHO Option B+ policy to prevent vertical HIV transmission (PMTCT). This paper is a sub-analysis of qualitative data from a mixed-methods multi-level process evaluation of Option B+ implementation in South Africa (SA). In this study we explored health facility users’ infant feeding knowledge, perceptions, and practices one year after SA adopted the 2016 updated World Health Organization prevention of mother-to-child transmission of HIV Option B+ infant feeding guidelines.

**Methods:**

Nineteen focus group discussions (FGDs) were held with six groups of men and women whose infants were aged < 6 months. Participants were attending randomly selected primary health care facilities within six purposively selected priority districts. The six groups included in the FGDs were: (i) adolescent girls and young women living with HIV (WHIV), (ii) adolescent girls and young women not living with HIV (WNHIV), (iii) older postnatal WHIV (iv) older postnatal WNHIV (v) pregnant women, and (vi) men. Data collection took place between April and December 2018. Data analysis involved coding and thematic framework analysis.

**Results:**

Women and men have suboptimal knowledge of the recommended breastfeeding duration and exclusive breastfeeding, especially for HIV-exposed infants. Most women received sub-optimal infant feeding counselling and mixed messages from health care workers. Fewer WHIV initiated breastfeeding at birth compared to WNHIV. Most parents believed that HIV-exposed infants should be breastfed for 6 months and many postnatal women on antiretroviral drugs and younger mothers lacked confidence to breastfeed beyond 6 months. Mixed feeding was predominant among all women due to individual, family, and socio-structural barriers. Many men were supportive on infant feeding; however, they lacked the appropriate information and skills to influence their partners’ infant feeding decisions.

**Conclusions:**

Differences in breastfeeding practices between WHIV and WNHIV are highly influenced by the lack of knowledge of infant feeding policy recommendations. Multiple-level factors deter many mothers from adhering to recommended guidelines. Appropriate ongoing infant feeding counselling and breastfeeding support are required for women and their partners.

## Background

In 2017, approximately 180,000 children were newly infected with HIV globally [1]. Many of these infections were vertically acquired during pregnancy, delivery, and breastfeeding. How children are fed during the first 1000 days of their lives influences their growth, development, and survival [2, 3]. The benefits of breastfeeding as a life-saving intervention [5–7] and exclusive breastfeeding (EBF) for the first 6 months as the best nutritional start for all infants are well documented [8–10]. Optimal breastfeeding practices reduce the prevalence of child morbidities and protect against other forms of malnutrition, including childhood obesity [11], to which younger non-breastfed infants are prone [12]. HIV acquisition through breastmilk and under-five mortality from all causes have been reported to drop when women living with HIV (WHIV) exclusively breastfeed from birth while adhering to antiretroviral therapy (ART) [13–15].

Given that ART greatly reduces the risk of HIV transmission during pregnancy and breastfeeding, the World Health Organization (WHO) and the United Nations Children’s Fund HIV and infant feeding update strongly recommends EBF for the first 6 months of life, and continued breastfeeding for up to two years or longer, with ART adherence support [9]. Further, it suggests that women practicing mixed feeding should not stop breastfeeding in the presence of antiretroviral (ARV) drugs. The South African National Department of Health (SA NDoH) adopted this guideline in July 2017 [9, 16]. Despite many interventions to promote optimal infant feeding practices, multi-level factors deter mothers’ ability to adhere to these recommended practices [17–19] resulting in early breastfeeding cessation or mixed feeding during the first 6 months, especially in low- and middle-income countries (LMICs), in Sub-Saharan Africa (SSA) [20, 21]. A review conducted in LMICs in 2016, showed that in SSA, only 37% of infants younger than 6 months were exclusively breastfed [7]. In Australia, a qualitative study found that 50% of mothers did not know the recommended age for introducing solids, and introduced solids around the mean age of 4.2 months [1.3–7.5 months] [22]. Many studies have reported on mothers receiving conflicting infant feeding messages not consistent with current guidelines [22–24]. In rural Rwanda, a qualitative study found that most respondents were aware of the WHO infant feeding recommendations, and rarely practiced mixed feeding from birth until 6 months of age. Authors attributed sustained and longer duration of EBF to increased infant feeding awareness due to health center and community-based education [25].

Infant feeding practices occur within the wider family, socio-cultural, economic, and health care system contexts. A systematic review by Niewoudt et al., found that at household level, supporting mothers after HIV status disclosure promotes EBF while gender norms and power relations undermine it [26]. Previous studies found that fathers saw the process of feeding infant expressed breastmilk and bottle feeding as opportunities for bonding with their babies [27, 28]. The positive effects of involving fathers in supporting partners on infant feeding have been previously reported [29].

The fathers’ knowledge of recommended infant feeding practices is associated with improved infant nutritional status [30]. In addition, the rates of early initiation of breastfeeding and EBF in many settings improved when locally-driven interventions were introduced [31, 32].

Antenatal HIV prevalence rates in South Africa (SA) remain high at 30.7% [33]. There is evidence that in SA young people face many barriers accessing sexual and reproductive health interventions at public sector health care facilities [34, 35]. The South African National Strategic Plan for HIV, STI’s and TB (2017–2022), seeks to develop and implement combination interventions for the prevention of new HIV and sexually transmitted infections [36]. The latest South African Demographic and Health Survey (SADHS) reported a national EBF rate of 32% for infants under 6 months of age [37]. Previous studies in SA have shown that some infants are not breastfed from birth [38], are given pre-lacteal feeds [39] or other feeds concurrently with breastmilk before 6 months, [40] and up to 40% of WHIV stop breastfeeding before 12 weeks [41]. Some of these sub-optimal feeding practices have been associated with vertical transmission of HIV [42, 43].

For almost two decades, the SA NDoH has introduced many changes to promote optimal infant feeding. These include adopting the ‘breastfeeding only’ infant feeding strategy [44], the 2011 Tshwane Declaration of Support for Breastfeeding, withdrawing the provision of free-formula milk as part of the programme to prevent mother to child transmission of HIV (MTCT) and encouraging health workers to promote EBF [45]. In addition, the 2013 Infant and Young Child Feeding (IYCF) policy [46] recommended breastfeeding for 12 months among WHIV and discouraged mixed feeding. In 2017 the 2016 updated WHO guideline was adopted [9, 16] and Breastfeeding Plus Counselling (an integrated nutritional, nurturing and medical intervention for mothers of HIV-exposed and unexposed babies) was introduced [47]. These changes, if not properly communicated, are likely to cause confusion among health care workers (HCWs) and health facility users.

To our knowledge, few studies have explored infant feeding perspectives from a variety of health service users in the context of WHO Option B+ policy to prevent vertical HIV transmission (PMTCT). We sought to explore health facility users’ perspectives on infant feeding practices in different settings in SA during the Option B+ era and one year after SA adopted the updated WHO infant feeding guidelines in 2016.

## Methods

### Data collection

Study data were collected as part of a mixed-methods, multi-level process evaluation of Option B+ implementation conducted approximately three years after Option B+ was adopted as national policy in SA. One of the objectives of the wider study was to identify focused solutions to key bottlenecks in the implementation of the ‘test and treat strategy for pregnant and lactating women’ and their families. It applied both qualitative and quantitative data collection methods. This study was conducted in randomly selected facilities within six purposively selected priority districts: OR Tambo (Eastern Cape province), Ekurhuleni (Gauteng province), eThekwini (KwaZulu-Natal province), Greater Sekhukhune (Limpopo province), Ehlanzeni (Mpumalanga province), and Bojanala (North-West province). These districts fell within the group of 25 districts with the highest number of HIV polymerase chain reaction (PCR) positive infants in 2014 and 2015 [48].

We planned to conduct focus group discussions (FGDs) among six different categories of purposively selected health facility users within each of the 6 randomly selected priority districts totaling 36 FGDs: i) adolescent girls and young women living with HIV (AGYWHIV) (aged 24 years and younger), ii) adolescent girls and young women not living with HIV (AGYWNHIV), iii) older WHIV (aged > 24 years), iv) older WNHIV, v) pregnant women regardless of age and HIV status and vi) men. All respondents should have had babies aged 0–6 months and should have been living in the district for > 6 months. The categories of participants for the wider study FGDs are listed in Table 1 and that of participants successfully interviewed in this study are in Table 2.

All women were recruited from antenatal, postnatal, and well-baby clinics and should have been either primigravid, para gravida, primiparas or multiparas. We planned separate FGDs for adolescent girls and young women (AGYW) since the Joint United Nations Programme on HIV/AIDS (UNAIDS) reported that in 2017, AGYW aged 15–24 years constituted only 10% of the population in SSA, however, they accounted for one in five new HIV infections [49]. FGDs for men (referred to as male partners) and pregnant women were not separated according to HIV status and age groups. This was according to the design of the wider study. For men, we anticipated that men rarely visit maternal and child health care services and might not be willing to disclose their HIV status. Men with infants below 6 months of age were therefore conveniently sampled from any of the health services offered within a primary health care facility.

Additional infant feeding data were collected from HCWs and mothers using in-depth interviews and structured surveys, respectively (findings will be reported in a separate paper).

Between April and December 2018, two experienced qualitative researchers (VM and DFN) conducted the FGDs using semi-structured topic guides (Appendix B) translated from English to isiZulu, isiXhosa, Seswati, SeSotho and Sepedi languages. In Limpopo, Mpumalanga and Gauteng provinces researchers sought the services of local interpreters to assist during FGDs. Semi-structured questions included respondents’ knowledge, views and practices about the new infant feeding policy. Specifically, we explored the following: i) knowledge of the recommended breastfeeding duration, ii) EBF practices, iii) breastfeeding and MTCT, iv) perceived quality of HCW counselling, v) male partner support, vi) other important breastfeeding-related issues, vii) contributory factors to inappropriate feeding practices, and viii) coping strategies. FGDs aimed at free sharing of experiences, in-depth exploration of issues without missing nuances within each category. FGDs lasted between 45 and 60 minutes. Researchers held regular meetings and debriefing sessions to critically explore participant responses, review field notes, discuss deviant cases and agree on codes and emerging themes until data saturation was reached.

We aimed for the standard size recommended for a focus group (i.e., between 6 to 10 participants) and invited more participants, however, fewer AGYW, WLHIV and men were enrolled. Consequently, two postnatal AGYWHIV recruited agreed to be combined with three older postnatal WHIV in one FGD in KwaZulu-Natal. However, responses from each category were labelled separately in the analyses. Experiences and views of the two AGYWHIV were explored in detail. In three FGDs, 5 participants were available and in one FGD only 4 participated. Male respondents were not necessarily partners to women who participated in FGDs.

### Data analysis

An independent service provider translated all audio- recordings into English and transcribed them using Microsoft Word. After translation, study researchers and team members fluent in the local languages of respondents read the transcripts and cross checked them against the audio-recordings to confirm the accuracy and consistency in translation and correct errors.

Data analysis was guided by grounded theory and a thematic framework approach. This involves researchers constructing a theory as an outcome of their interpretation of the participants’ stories. Researchers generate a general explanation of a process, action, or interaction shaped by the views of many participants. Themes are identified inductively from the views and experiences of research participants. Extensive and varied raw text data are condensed into a brief summary format, clear and justifiable links established between the research objectives, and a theory/model developed about the underlying structure of experiences or processes in the text [50–53].

Transcripts were also read several times to develop familiarity with the data. Transcripts were independently coded by two researchers and later compared for accuracy. The expert qualitative researcher (MG) reviewed the same transcripts, and where the coding by two researchers differed, this was discussed until consensus was reached. Data including illustrative quotes were identified and charted into the thematic framework [51] which was further discussed and refined under the guidance of the expert consultant. The study team periodically reflected on identified themes, sub-themes, and illustrative quotes to resolve any discrepancies.

## Results

We conducted 19 FGDs with total of 113 respondents falling into the following categories (i) postnatal older women not living with HIV (WNHIV), ii) postnatal older women living with HIV (WHIV), iii) postnatal adolescent girls and young women not living with HIV (AGYWNHIV), iv) pregnant women and v) men. Eight main themes, and seventeen sub-themes emerged from the data (Table 3). As a guide to quantify qualitative findings and to ensure consistency, we applied the framework (Table 4) to describe the meaning of ‘most’, ‘many’, ‘some’, ‘few' by estimating the proportion of respondents who reported similar answers [54].

### Infant feeding themes

#### Knowledge and practices about the recommended breastfeeding and breastfeeding duration

Most respondents including men understood the benefits of breastfeeding and preferred their infants to be breastfeed, however, not many intended to breastfeed for two years and beyond and few WHIV reported initiating breastfeeding at birth compared to WNHIV.

*“I prefer breastfeeding because I didn’t breastfeed my first baby, so with this one, I want nothing but to breastfeed for one year, six months.”* Pregnant woman (North- West)                                                                                                                                                                                               

*“They say breastmilk is good for a baby. As a father, especially an African, feel happy when I see my baby being breastfed because I know she will grow well. They say breastfeeding also prevents sicknesses...”* Male partner (Mpumalanga).

*“I just decided to feed my baby formula. So, I started her on formula …*” Postnatal WHIV (Mpumalanga).

We asked about the expected duration of breastfeeding. In all districts, most respondents believed HIV-exposed babies should breastfeed for a shorter duration of 6 months to minimize the risk of vertical HIV transmission, asserting that they received this advice from HCWs. Some women tended to confuse the duration recommended for breastfeeding with that for EBF.

*“[At the clinic] We are told that you can breastfeed and stop when the baby is 6 months old because breastmilk is clean.”* Postnatal WHIV (KwaZulu-Natal).

*“They [WNHIV] should breastfeed for 2 years, that’s what the counsellor said. They say sometimes it can be up to 5 years, but if you can breastfeed for at least 2 years, it is still better.”* Male partner (Mpumalanga).

*“They [WHIV] must breastfeed for the stipulated time, which is 6 months; after that I do not know. After that they can then use the bottle. HIV positive mothers can breastfeed until 6 months … [thereafter] give food and stop breastfeeding.”* Postnatal WNHIV (Mpumalanga).

The importance of resuming breastfeeding even after other feeds have been introduced or after interrupted breastfeeding was unknown to a majority of respondents; the majority were over-concerned about the dangers of mixed feeding.

*“I breastfed her for 3 months, because I used to knock off from work very late around 12h00 midnight or at 04h00 in the morning. I then stopped breastfeeding and started formula feeding … No [ I did not continue breastfeeding], because I thought it was not going to be good for her to breastfeed her only when I come back home late at night.”* Postnatal WNHIV (Mpumalanga).

Although many male respondents were clear about the recommended duration of breastfeeding, some expressed that their power to influence to their partners’ decisions was limited.

*"As husbands, our challenge is that even though we would like our babies to be breastfed for 2 years, if the mother has decided to stop breastfeeding, you can’t force her; instead, you try and support what she prefers … there are sometimes challenges such as breast lacerations which then make 2 years [of breastfeeding] longer.”* Male partner (Mpumalanga).

#### Exclusive breastfeeding knowledge and practices

Almost all respondents were aware of the first six months EBF recommendation, however, many women felt adhering to the EBF recommendations was difficult and impractical. As a result, most women resorted to introducing other feeds very early to address perceived infant feeding problems. These women strongly felt that practicing EBF was not as easy as it was made to look.

*“We know that we should not mix-feed until the baby is 6 months old. But we are the ones experiencing challenges. What is written on paper does not always work. We are the ones who are taking care of our babies, and when we give the baby other feeds, the babies’ problems get resolved.”* Postnatal WNHIV (Eastern Cape).

*“At the hospital we are told to breastfeed for 6 months and not to mix-feed especially if one is HIV positive. You should either give breastmilk only and no other feeds or formula milk only.”* Postnatal WHIV (KwaZulu-Natal).

Some women stated that they practiced EBF as they were aware of the dangers of mixed feeding for both HIV-exposed and HIV-unexposed infants.

*“[I feed her] breastmilk. She is 3 months old and I have not started giving solid foods. I am exclusively breastfeeding [and will breastfeed] until 2 years. I will start giving solids when she is 6 months old because I want the baby to grow well.”* Postnatal WNHIV (Mpumalanga).

*“They [HCWs] say it is risky to mix-feed, giving the baby breastmilk and formula. You should use one method of feeding because this can create difficulties for the baby, because it is not easy for the baby’s body to adjust … The baby’s’ stomach gets used to one pattern of feeding or else she could suffer pain in the stomach or have diarrhea or feel heaviness in the stomach due to mixed feeding.”* Postnatal WNHIV (Eastern Cape).

Many WHIV from different districts stated that they did not initiate breastfeeding despite knowing that they could breastfeed without transmitting the virus to the baby because they were taking ARVs. Among those who initiated breastfeeding, most either intended to stop breastfeeding at 6 months or had already stopped.

*“I just decided to feed my baby formula. So, I started her on formula … Another reason is that I will be going back to work. I thought it would be challenging to stop breastfeeding. … Yes, I believe [an HIV positive mother] can breastfeed the baby and that a baby can remain uninfected.”* Postnatal WHIV (Mpumalanga).

*“I was still enjoying breastfeeding my baby. I didn’t clearly understand but I heard that if I introduce porridge early her stomach will be irritated, so I stopped [breastfeeding].”* Postnatal WHIV (Gauteng).

While most of respondents were aware that mixed feeding before 6 months is discouraged, some thought mixed feeding was contra-indicated only among HIV-exposed babies but carried no risk for HIV-unexposed babies.

*“I know that you can breastfeed your baby until 6 months old even if you are HIV positive and the baby will not be infected because after delivery your baby comes out with your blood cells. Therefore, it is very important that you if you are HIV positive, you don’t mix feed by giving both breastmilk and formula because that can make your baby to be HIV positive.”* Postnatal WNHIV (Eastern Cape).

*“They [HCWs] told me if you are HIV positive, you are not expected to mix-feed, when the child has not reached 6 months.”* Postnatal AGYWHIV (KwaZulu-Natal).

One woman, however, displayed knowledge that mixed feeding before 6-months carries other risks for all babies, besides HIV transmission.

*“It is because those babies are still too young for solid foods. If you cook porridge for the baby, then the baby will have an upset stomach. Then you will go to the clinic complaining that your baby is constipated and crying a lot …*. *Their intestines are still very tiny to carry solid foods. That is why we should only give them [breast] milk only. After 6 months, when he has grown up a bit then you can then start giving solid foods, but not so many spoons. You can feed him 3 teaspoons then give formula.”* Postnatal WNHIV (Mpumalanga).

#### Breastfeeding and mother-to-child transmission of HIV (MTCT)

Although many women confirmed receiving useful information from the clinics about breastfeeding as a mode for vertical transmission of HIV, some misunderstood how this occurs. This lack of clarity influenced their decisions to continue with breastfeeding.

One WHIV told us she could not breast all her babies because she had previously been diagnosed with cancer on one breast.

 "*Yes, I do believe that you can breastfeed your baby and not infect her with HIV, they do tell you how long you can breastfeed. I couldn’t breastfeed all my babies because I once had cancer, so I had to use only one breast.”* Postnatal WHIV (Mpumalanga).

##### Fear of infecting the baby

Most WHIV expressed fears of transmitting the virus to their babies if they continued breastfeeding babies beyond 6 months. A majority of respondents, including WNHIV and men, strongly believed that HIV acquisition from breastfeeding only occurred when older babies bite and suck infected blood from cracked nipples.

*“At 6 months when the baby’s teeth start to develop, he may bite the breast and swallow infected blood, so it is not safe for the baby [to be breastfed].".* Pregnant woman (North-West).

*“I don’t think they [WHIV] should [breastfeed] … The reason being that the baby may bite and swallow her blood, and then get infected.*” Male partner (Mpumalanga).

One adolescent WHIV on ARVs shared how her fears to infect her baby caused her to stop breastfeeding after few days.

*“I was very scared to breastfeed because I do not believe that the baby can be fully protected [by ARVs] ... I kept thinking about cracked nipples, getting bitten by the baby and about what to do should the baby get infected? I only breastfed when I was still in hospital; after discharge I stopped.”* Postnatal AGYWHIV (KwaZulu-Natal).

When asked if HIV can be found in the breastmilk of a WHIV, most women were adamant that the virus is found in blood and not in breastmilk; and few were unsure.

*“No, the breastmilk does not have it [HIV virus] … I know so many people in my neighborhood who breastfed their babies while HIV positive, but their babies are not infected.”* Postnatal WNHIV (Eastern Cape).

*“[At the clinic] We are told that you can breastfeed and stop when the baby is 6 months old because breastmilk is clean. ARVs clean the system and not breastmilk. Breastmilk is separate and not contaminated.”* Postnatal WHIV (KwaZulu-Natal).

 "*Mmh … I am also not sure if breastmilk can infect the baby or not*.” Postnatal WNHIV (Eastern Cape).

##### Effectiveness of ARVs to prevent HIV transmission through breastfeeding

Respondents gave mixed responses when asked if they believed that breastfed babies are protected from HIV infection when their mothers are on ARVs. Despite many knowing that ARVs are protective, most women, particularly WHIV, were skeptical about breastfeeding and did not feel confident breastfeeding for longer than 6 months due to fear of infecting them.

*“I believe that the baby may not be infected when you are taking ARVs, but I sometimes don’t believe that the baby can be completely safe because while breastfeeding you can develop an abscess … , although there is medication given to the baby during breastfeeding.”* Postnatal WHIV (Mpumalanga).

"*Yes, they can. HIV positive mothers can breastfeed a baby, but for a limited time of six months. It is safe because the mother will be taking their ARV treatment, the infection won’t pass to the baby*.” Pregnant woman (North- West*).*

##### Challenges with anti-retroviral therapy adherence

In few districts, some WHIV were concerned about ARV stock-outs and complained that sometimes they do not get their ARVs on the same day when coming for baby immunizations, due shortage of personnel and poor service integration.

*“If you are collecting treatment, you do not always get it. For instance, it happened twice that I had to come for treatment because of staff shortage … If you brought the baby for immunizations today and the doctor needs to go to maternity ward, you won’t get ARVs. You will need to come back tomorrow because the doctor must deliver someone at the labour ward. … You are turned away and will be told to arrive at 05h00 tomorrow so that you don’t join the queue.”* Postnatal WHIV (Mpumalanga).

In most districts, most postnatal WHIV denied ‘ever missing their ARVs’ but many said they rarely took them on time every day.

*“I swallow them [ ARVs] as soon as I get home … Sometimes I think I will be home before nine but end-up arriving late … I don’t ever sleep without swallowing them. If I am not sure if I will be home before nine, I wrap them in a tissue and put them in my handbag.”* Postnatal WHIV (Mpumalanga).

##### Poor viral load monitoring

In one district, some breastfeeding WHIV shared their frustrations about the failure of some clinics to monitor their viral loads during breastfeeding due to shortage of laboratory supplies. They reported that this affected their confidence to continue breastfeeding.

*“[My viral load] should have been checked [last month but the blood tubes were expired … I’m breastfeeding and need to know if my viral load is suppressed or not so that I don’t put my baby at risk... Now I’m always worried, thinking about what could happen if it is not suppressed.”* Postnatal WHIV (Mpumalanga).

##### HIV status disclosure

Few WHIV mentioned that fear of HIV stigma and discrimination affected their infant feeding practices.

“*I fear [disclosing my HIV status] that he may not be supportive … I just don’t know how to approach him because he does not speak well about HIV positive people, you see*.” Post-natal WHIV (Mpumalanga).

Many WHIV reported that disclosing their HIV status to family members was beneficial.

"*I use my phone [as a reminder to take ARVs], but also my family is very supportive. At seven o’clock they bring ARVs for me to take … My mother, sisters and my kids remind me*.” Postnatal WHIV (Mpumalanga).

#### Perceived quality of counselling

##### Health care worker counselling

While almost all women acknowledged HCWs as an important resource for infant feeding information, many complained that they did not receive relevant infant feeding counselling. A majority of WNHIV felt that post-test counselling was sub-optimal and did not address their specific needs.

*“I got tested and I was told that I am HIV positive, but I did not receive counselling. They asked me how I feel; then they told me that I will be given an injection to protect me from getting TB because I did not have TB and will then start treatment [ARVs] …. Later, I joined Mom Connect.”* Postnatal WHIV (Mpumalanga).

Many WNHIV postnatal women, especially inexperienced and younger women felt that counselling sessions mainly focused on how to prevent HIV transmission through breastfeeding and very little attention was given to addressing other topics such as lactation management.

*“I was only taught about breastfeeding if you are HIV positive. I was not taught how to breastfeed and how to hold the breast …”* Postnatal AGYWNHIV (KwaZulu-Natal).

While many mothers complained about poor HCW attitudes which they described as a barrier to effective counselling, some appreciated HCW efforts in addressing their infant feeding information gaps.                                                                                                                                            

*“It depends on the nurses that you deal with. Sometimes they treat you well. Even if you ask something they respond well. But sometimes if you ask something they do not answer you or they will tell you that they do not know because they are rotating their duties as staff or they have staff shortages."* Postnatal WHIV (Mpumalanga).

*“Sometimes they scold us saying children will have tooth decay because of bottle-feeding … When you come to the clinic with a pacifier, they throw it away.”* Postnatal WNHIV (Gauteng).

Many pregnant women, however, acknowledged the counselling and on-going group educational sessions provided during antenatal care (ANC) and viewed them as informative.

*Every morning, there is an education session for pregnant women. They educate us about different topics like HIV and breastfeeding. I think this is very good.”* Pregnant woman (Eastern Cape).

*“The counselling was fine; ... very good counselling; they counselled about everything, and they did the [HIV] test, so I was satisfied*. Pregnant woman (North-West).

##### Accuracy of infant feeding messages

Most respondents expressed dissatisfaction with the content of HCW counselling messages stating that they were inconsistent and sometimes contradictory. They highlighted that no explanation was given about reasons for the infant feeding policy change.

*“[One nurse] said it was not right to formula-feed and that the milk would come out if I continued to put her onto the breast. I felt pressured here at the clinic when they told me to breastfeed and stop formula feeding. So, later, I decided to stop breastfeeding because they [HCWs] had told me to exclusively breastfeed. During my next clinic visit, a different nurse asked me why I had stopped breastfeeding … … She said mixed- feeding is fine, there is no problem with it, I can feed her both. So now, I don’t know, the two nurses were saying opposing messages …”* Postnatal WNHIV (Gauteng).

Two young WHIV explained how they had to change their intended infant feeding practices to align with what they had been told at the clinic.

*“I am also going to stop breastfeeding at 6 months. I was also told [at the clinic] that the child might get infected if I go beyond 6 months; but I would have loved to carry on.”* Postnatal AGYWHIV (KwaZulu-Natal).

*“The sister said I should not breastfeed beyond 6 months because there is a possibility that my baby will be infected. So, I was told to stop at 6 months.”* WHIV (Limpopo).

##### Experiences with mom connect

Many mothers appreciated being registered with the SA NDoH’s Mom Connect program [55] and deemed messages received as relevant and informative. Most felt that Mom Connect helped to bridge some of the knowledge gaps they had.

*“For me, Mom Connect feels like a home-based visit by the clinic staff because of the information. Messages come every week; maybe three or four times a week, advising me what to do when my child was responding in a certain way.”* Postnatal WHIV (Gauteng).

*“I did not receive counselling, but I joined Mom Connect immediately because I had just started taking [antiretroviral] treatment. … You get most information from Mom Connect.”* Postnatal AGYWHIV (KZN).

#### Lack of infant feeding / breastfeeding support

Respondents were asked about their views and experiences about roles of HCWs, male partners and families on infant feeding or lactation support.

##### Support by health care workers

Most inexperienced breastfeeding mothers complained that they had to seek advice from family members when facing breastfeeding complications as most clinic staff could not provide on-going practical breastfeeding support.

“*I did not know how to breastfeed the baby. I did not know how to hold the breast. … My grandmother helped me. The clinic staff did not help me.”* Postnatal WNHIV (Gauteng).

*“I had to stop breastfeeding one month after birth because my breasts were full and heavy, … I had a lot of breastmilk and experienced breast engorgements while breastfeeding, and the baby was crying continuously.”* Postnatal WNHIV (Eastern Cape).

##### The role of male partners

Many female respondents narrated the difficulties experienced due to lack of male partner support. They reported that the absence of financial, practical, and emotional support from their partners affected their infant feeding intentions and practices.

*“It’s hard to be a teenage mother. I don’t sleep at night because the baby sleeps mostly during the day. I sometimes cry because I don’t have someone to help me with the baby. He [partner] always says he is busy …. I sometimes drink energy drinks at night to stay awake so I can take care of the baby.”* Postnatal AGYWNHIV (KwaZulu-Natal*).*

*“As for money! We would fight even when we have to buy pampers. He would say he does not have money, but he is working. I don’t understand, he would always say he does not have money every time you ask money for something. He would give you money once in a while, …*.” Postnatal WNHIV (Eastern Cape).

Many men had brought their infants for immunization or minor ailments which showed they were providing some practical support to their partners for childcare.

“*I have noticed is that when they [women] initiate breastfeeding, their breasts become painful. Therefore, I need to go buy medication for cracked nipples because I can see that she is really struggling. She is keen to breastfeed but it’s painful. She needs to apply something so that she can continue breastfeeding*.” Male partner (Mpumalanga).

Although many men asserted that they were providing both financial and emotional support by assisting to resolve many infant feeding challenges their partners were facing, some of these infant feeding practices were not in line with the policy guidelines.

*“I had hoped [that she will be breastfed] for 2 years. She is only 2-weeks old, but we have already bought tablets to stop breastmilk production. Tomorrow is her last day of breastfeeding because her mother is a student. I will be taking my child to my girlfriend’s mother in Giyani.”* Male partner (Mpumalanga).

*“My baby was breastfed from birth. She was 2 months and not getting full from the breastfeeding only. As a result, her mother asked me to buy formula. So now she is getting both breastmilk and formula and sometimes we give her ‘baby porridge’ and then she stops crying.”* Male partner (KwaZulu-Natal).

Most men confirmed that they did not have accurate information about infant feeding because they rarely visited the clinics where they believed they could get counselling.

*“I don’t know much [about infant feeding] because I don’t visit the clinic often since I am working, but because I took a day off today, I decided to bring my kids to the clinic.”* Male partner (KwaZulu-Natal).

#### Other breastfeeding- related challenges impacting on HIV transmission through breastfeeding

##### Unknown partner HIV status

Most women highlighted several challenges that impact on infant feeding decisions and practices. A majority of WNHIV viewed themselves to be at high risk of acquiring HIV and transmitting it vertically through breastfeeding as most articulated that they were unaware of their partners’ HIV status as their partners were reluctant to test for HIV.

*“I do not know his status … He just says I must go and get tested and that if my result is negative, it means he is also negative.”* Postnatal WNHIV (Gauteng).

This reluctance to test was also confirmed by many male partners who displayed lack of understanding about HIV discordancy.

*“Because we are scared to visit the clinic for [HIV] testing, so if a woman has tested negative, then I know that I am also negative.”* Male partner (Mpumalanga).

*“I got tested [for HIV] after delivery, but her father was not tested. He does not want to come for testing, he is always busy … , he says I should go get tested alone; he is not part of that arrangement.”* Postnatal AGYWNHIV (KwaZulu-Natal).

##### Inconsistent or non-condom use

Many WNHIV expressed concerns about their partners’ unfaithful behaviors’, including non-condom use. Some women explained how their male partners instilled guilt and manipulated them into agreeing to engage in condom-less sex during the breastfeeding period.

“*[He] will ask: ‘Why are you telling me to use this [condom] now? … If you want to be a good wife, you won’t make me use condoms because you trust me and I trust you, right?’ So, I end up allowing him to remove the condom.”* Postnatal WNHIV (North- West).

Many male respondents also confirmed engaging in unsafe sex and not knowing their HIV status. One man stated that his decision to use condoms was based on whether the woman looked attractive or not.

*“When she is too pretty and looking great, I just tell myself that I’m not going to use a condom.”* Male partner (Mpumalanga).

##### Poor HIV risk perception

Two WNHIV from Gauteng strongly believed they were not at risk of acquiring HIV despite having a history of non- condom use and alcohol use.

“*[I feel] 100% safe although I do not use a condom. The day he suggests we should use a condom, then I will suspect that something is wrong because we have never used condoms in the last eight years.”* Postnatal breastfeeding WNHIV (Gauteng).

“*I trust my partner … ; and he trusts me too. We do not use condoms and there is no [HIV] risk. Even when we drink, we both drink together in the house and have fun.”* Postnatal WNHIV (Gauteng).

#### Other factors contributing to inappropriate infant feeding practices

Besides HIV-related factors, respondents cited other challenges which prevented them from adhering to the recommended infant feeding practices. These included perceived insufficient milk and infant cues, schooling and employment, family pressures and maternal attitudes to breastfeeding.

##### Perceived insufficient milk and infant cues

Most mothers told us they felt compelled to introduce formula early because they thought that babies were not getting full, or breastmilk was insufficient.

*“I tried to breastfeed, but I could see that she was not getting full. I ended up buying formula and stopped breastfeeding immediately, 4 days after discharge from hospital.”* Postnatal WHIV (KwaZulu-Natal).

*“Yes, I am giving her formula and I have just started feeding her maize porridge … [She is] 10 weeks old but she is not getting full, she finishes a big bottle and sleeps for a short period of time then wake up crying. I think I am going to add XXX porridge once she is 3 months old.” *Postnatal WNHIV (Eastern Cape).

*“My milk is no longer enough. She sleeps for two hours and wakes up hungry. Every two hours she wants to be fed. I cannot manage.”* Postnatal WNHIV (Gauteng).

##### Schooling and employment

Most women stated that the need for schooling and employment often forced them introduce formula earlier than 6 months or to stop breastfeeding early. Many mothers did not know that they could express breastmilk to feed their infants during their absence and that they could resume breastfeeding even after they had introduced other feeds.

*“I feed her XX [infant formula] because I am a student. When I am at school, they feed her formula and when I come back, I breastfeed. I have a lot of milk.”* Postnatal WNHIV (Gauteng).

When mothers were asked if they left expressed breastmilk for their infants, most reported they discarded it.

*“Sometimes the breasts are sore, and they leak. I usually go to the toilet to squeeze all the milk out. I had to introduce it [formula] because the child must eat during the day when am not home.”* Postnatal WNHIV mother of a 6- weeks old infant (KwaZulu-Natal).

*“I breastfeed when I am home, but they feed her formula when I am at school.” *Postnatal WHIV (Eastern Cape).

One man explained how he was supporting his partner to wean their newborn baby due to schooling demands.

*“I support [breastfeeding for] 2 years; but because she is working. She breastfed for a week and stopped because she had to go back to work” *Male partner (Mpumalanga).

##### Family pressures

While many mothers considered family members, especially infants’ grandmothers, as important sources of information, younger mothers felt pressured to comply with instructions from older family members.

*“At home they started pressuring me to introduce other feeds as soon as the child’s umbilicus fell-off. My child started solids when she was 1 month old”* Postnatal WNHIV (Gauteng).

*“They [mother and sister] put pressure saying the baby is crying, therefore it means she does not get full … If you try to argue with an older person, they will tell you that you are disrespectful ….”* Postnatal WNHIV (North-West).

##### Maternal attitudes to breastfeeding

Some men expressed that their partners preferred not to breastfeed due to concerns about their physical appearance and others expressed they felt powerless to persuade their partners to breastfeeding for longer periods.

*“Ladies do not want to breastfeed; they do not want sagging breasts, you see. Other women formula-feed just to show-off to friends that they can afford to buy formula. … Most worry about exposing their breasts [during breastfeeding]”.* Male partner (Mpumalanga).

#### Coping strategies for mothers

Some experienced mothers who had been successful in practicing EBF shared how they dealt with and coped with pressures to mix-feed and other infant feeding problems.

*“You should not be instructed by someone not to exclusively breastfeed. It is your choice. If you want to go back to work, you may continue and only breastfeed at night. No one should force you to stop. … At home, they told me to feed her porridge, but I was stubborn. I refused and told them that I will breastfeed until 6 months.”* Postnatal WNHIV (Gauteng).

*“Sometimes she was not getting full, but then I would breastfeed for longer”.* Postnatal WNHIV (Limpopo).

Both women and men thought that joining support groups would empower them to cope with infant feeding challenges. They also felt that HCWs should educate men on infant feeding.

*“I think support groups for mothers can be useful. In our meetings we can talk about how to keep our infants’ HIV negative, we can also empower one another.”* Postnatal WHIV (Mpumalanga).

## Discussion

This qualitative study was conducted in SA almost a year following the adoption of the updated 2016 WHO HIV and Infant feeding guidelines which made infant feeding recommendations universal for both WHIV on ART and WNLWH [9]. We conducted FGDs with different categories of health facility users and found that, while most participants were aware of the benefits of breastfeeding, many did not comply with several recommended infant feeding guidelines due a combination of individual, socio-cultural, and health system factors. A systematic review of different infant feeding policy influences on EBF in SA similarly found that factors interacted across multiple levels of influence. However, most of these studies included in the review fell short in accounting for family, community and workplace influences on EBF [26]. Our study filled this gap by exploring issues around employment and family infant feeding challenges.

### Breastfeeding duration knowledge and practices

Our study found that most women and men were not aware that the recommended breastfeeding duration is universal for both WNHIV and WHIV, provided WHIV are virally suppressed and supported to take ARVs consistently. Although most respondents understood that infants of WNHIV can be breastfed for 2 years and beyond, a majority held strong views that WHIV should breastfeed for a less than 6 months to prevent MTCT. We also found that breastfeeding practices differed across WHIV and WNHIV with fewer WHIV initiating breastfeeding at birth compared to WNHIV. In line with what most respondents believed, most WHIV had already stopped or intended to stop before 6 months while many WNHIV intended to breastfeed beyond one year. Similarly, West et al., found that WHIV were less likely to continue breastfeeding longer than 6 months compared to WNHIV [24]. We found that some women tended to confuse the recommended duration of 6 months EBF with the expected duration of breastfeeding. These findings suggest that HCW counselling potentially continues to highlight the dangers of mixed feeding instead of the benefits of breastfeeding in line with the previous South African 2013 IYCF guideline which strongly emphasized EBF in the first 6 months and shorter breastfeeding duration for WHIV [46]. Our finding is also in line with the results from previous studies which reported poor maternal knowledge in relation to the recommended time for introducing other feeds and the duration of infant feeding [22, 56, 57].

Our study found that both short-term and longer-term breastfeeding duration remain a problem despite most women reporting that they initiate breastfeeding. In our study, some mothers had already stopped breastfeeding and introduced other feeds as early as the first week of the infant’s life. Previous data from SA showed that EBF rates for infants aged 4–8 weeks had increased, coinciding with the Tshwane Declaration of Support for Breastfeeding in SA [58]. We also found that some men knew the recommended duration of breastfeeding for WNHIV, however, many also believed that HIV-exposed infants should only be breastfed for 6 months. This lack of knowledge among health facility users is concerning, given that previous studies have positively associated awareness of the benefits of breastfeeding with safe infant feeding practices and consistent EBF [26, 59].

#### Exclusive breastfeeding knowledge and practices

Similar to findings from previous studies in SSA [57, 60, 61], we found that mixed feeding remains a predominant form of infant feeding in SA, regardless of the woman’s HIV status and social circumstances. Our finding that most mothers reported early introduction of other feeds before 6 months while breastfeeding are consistent with those of the SADHS which showed EBF rates of 44% among infants aged 0–1 month of age compared with 24% among infants aged 4–5 months [37]. Similarly, a prospective observational cohort found that significantly more HIV-positive mothers in SA practice EBF compared with those HIV-negative at 3 weeks and reported a significant drop of this proportion by 4 months post-delivery [62].

While previous studies found that WHIV and WNHIV may choose to mix-feed to avoid HIV stigma [63, 64], our results found that very few mothers were concerned with fear of HIV stigma when making infant feeding decisions. This finding could be related to considerable progress made on stigma-reduction interventions since 2002 in SA [65].

In line with previous findings (Kavle et al., 2017, Hazemba et al., 2016), our study found that early cessation of breastfeeding and mixed feeding are common among women who experience lactation problems, ill-health or lack confidence to breastfeed. In 2014, Ijumba et al., found that it was a social norm for grandmothers in SA to introduce other feeds before 6 months of age [66]. Women in our study felt that practicing EBF for 6 months was difficult and impractical due to individual, family, and societal barriers. Consistent with our finding, disadvantaged mothers in Australia [22] and most WHIV and WNHIV in SA perceived EBF for 6 months to be difficult due to cultural factors and family influences [59]. We found many AGYW often unwillingly surrendered their parental instincts and accepted inappropriate advice from older family members.

### Vertical transmission of HIV through breastfeeding

Our study also found that most respondents were not clear how HIV vertical transmission occurs through breastfeeding. Although most women believed they could safely breastfeed without transmitting the virus to their infants if consistently taking ARVs, many WHIV were skeptical about breastfeeding and tended to stop breastfeeding early post-delivery. Many respondents thought that the HIV virus is not found in breastmilk and believed that vertical transmission occurs when older babies suck infected blood from the breast lacerations. While most WHIV did not view breastmilk as a vector for HIV transmission [67, 68], it is unclear why WHIV were skeptical to breastfeed when most reported adherence to ART. It is likely that some were non-adherent to ART and were thus motivated by fear of transmitting the virus, as reported in previous studies [23, 24]. Kavle et al. (2017), found HIV stigma, non-disclosure of status, and fear of transmitting the HIV virus to the infants as barriers to optimal breastfeeding among WHIV [18]. These findings underscore the need for counsellors to use simple and unambiguous messages and to clearly explain reasons behind the new infant feeding policy recommendations [9].

Our study found that in some districts, health system related factors such as poor viral load monitoring, ARV stock-outs and poor integration of services were highlighted as possible influencing factor to optimal breastfeeding practices. This finding has been previously reported in studies in SSA [69–72].

#### HIV – related factors impacting on breastfeeding practices

Incident HIV infection among pregnant and breastfeeding women after a first negative antenatal test is the key driver of ongoing MTCT [73]. In our study, it was concerning to learn that some breastfeeding WNHIV did not perceive themselves to be at risk of contracting HIV, despite reporting non-condom use and alcohol use. We found that most men did not use condoms consistently and were reluctant to test for HIV. Consistent with our study, previous findings showed that men are significantly underrepresented in HIV testing and treatment services – both in SSA and globally [74]. Despite the introduction of a combination HIV prevention interventions for AGYW aged 10–24 years [75, 76] and significant progress made in expanding access to HIV testing in SA, HIV testing uptake has remained relatively low among men compared to females [77].

In the context of ANC, few males attend clinics with their partners to benefit from ANC/PMTCT services [78]. In our study most man lacked knowledge on how HIV transmission occurs through breastfeeding and attributed this to their infrequent clinic visits. Interventions to promote male partner/couple attendance at ANC facilities and HIV testing in Uganda and Kenya, respectively, improved male HIV testing uptake [79, 80]. Previous studies have found a consistent association between alcohol consumption and engaging in unprotected sex [81, 82], risks for HIV acquisition [83] and ART non-adherence [84]. These findings have implications for the achievement of the UNAIDS 90–90-90 HIV treatment targets [85] and highlight the need for a comprehensive approach to addressing infant feeding issues within communities.

### Other barriers to optimal infant feeding practices

Women cited several barriers influencing their decisions to practice optimal infant feeding. These include pressures from family members, work and schooling demands, perceived insufficient breastmilk, infant cues, lactation challenges and maternal ill-health. Many learners and working women experienced engorged breasts and many discarded expressed breastmilk instead of feeding their babies. These results highlight the need for breastfeeding counselling and support and for school- and workplace- based programs to create conducive environments for breastfeeding mothers [26, 86, 87].

### Health care worker counselling

Our study found that many women complained that HCW counselling was confusing, with contradictory messages and followed a didactic approach. We found that although a few women were aware that they could resume breastfeeding after interrupted periods, many did not know the rationale for resuming breastfeeding. Post-natal counselling for WNHIV was not related to their needs and some found that the negative attitudes of HCWs prevented them from asking questions. Many women, however, acknowledged receiving informative infant feeding messages through the Mom Connect programme. Previous studies in SSA have found that mixed messages which are inconsistent with guidelines [22–24] were due to frequently changing guidelines [23]. Prior to 2016, HCWs attending a training intervention update on infant feeding and HIV in SA confirmed they lacked information and skills prior to training [88]. Previous gains from the PMTCT programme in SA might be reversed due to a failure to tailor infant feeding counselling to individual needs. Nieuwoudt et al., suggests that rapidly adopting and introducing global guidelines to an unsupported health workforce might lead to unintended damage [26].

### Men’s involvement

The role of men as supporters to infant feeding practices has been explored in recent years [89, 90]. Similar to previous studies [91, 92] we found that most of our male respondents preferred their infants to be breastfed and took interest in how their babies were fed, often providing practical, material, and emotional support to their partners, however, the support was often contrary to the recommended infant feeding practices. We found that many men have limited powers to influence their partners to adhere to optimal infant feeding practices. Our finding about increasing involvement of men in infant feeding matters and their limited influence on mothers’ decision to breastfeed is consistent with those from a qualitative systematic review which indicated that it is rare for men to be active decision-makers on infant feeding [93].

### Limitations and strengths

Our study has several limitations. Four of the FGDs discussions deviated from methodological recommendations of appropriate focus group size. We aimed for the recommended number of participants (i.e., between 6 to 10 participants) but some FGDs had fewer numbers due to unavailability of eligible participants. Moderating the conversation in a too small or a too large group is more difficult and can negatively affect the productivity of the group (as not all may engage fully in the discussion) [94]. The variation in group size and categories may have negatively affected the depth and richness of the data. FGDs for pregnant women and male participants were not categorized by HIV status and age because these data were not available during the study interview. We therefore could not explore how HIV may have affected the breastfeeding perceptions and practices for these groups.

Our study was cross-sectional and therefore did not capture respondents’ changing perspectives and practices over time. Response bias could have been introduced due to self-reports, especially in relation to men’s role as supporters of breastfeeding since men were not partners of women who participated in FGDs. In all districts, few AGYW and men were recruited, however, among those interviewed, infant feeding perspectives from each category were explored in-detail until data saturation was reached. Where AGYWHIV were combined with older WHIV, their responses were clearly identified. One strength of our study is that we interviewed different categories of health facility users across various district settings to elicit a broader perspective and included both male and female users.

### Implications

Given the challenges of the high HIV prevalence [95], poor postnatal retention particularly in women who initiate ART during pregnancy [72], and poor viral monitoring in SSA [69–71], our study findings underscore the need for strengthening existing interventions aimed at eliminating MTCT. These findings show that infant feeding practices are socially embedded, therefore adopting broader stakeholder engagement is necessary when infant feeding policy decisions are made. Strengthening ART adherence support, programme integration and promoting pre-exposure prophylaxis as part of a comprehensive HIV prevention package is urgently required. In addition, proven innovative strategies for disseminating accurate infant feeding counselling messages to all family members, could enhance infant feeding practices. The use of community-based peer supporters [96], mobile technology [55, 97], and introducing innovative HCW training approaches [88, 98] have all shown promising results in bridging the knowledge and skills gaps among key stakeholders.

## Conclusion

Our findings highlight that in the context of Option B+ implementation, infant feeding remains complex and challenging, especially for women in SA. Despite the infant feeding policy change, there is still much misunderstanding and misinformation about infant feeding recommendations across different categories of health facility users resulting in sub-optimal practices. Conflicting messages, individual and social demands, and the level of maternal infant feeding support received play an important role in influencing mothers’ infant feeding decisions. Men are becoming involved and supporting their partners on infant feeding; however, they lack appropriate knowledge. Comprehensive strategies are needed to streamline infant feeding messages, promote male partner involvement, and improve mothers’ confidence to practice optimal infant feeding. Future prospective qualitative studies could help explore changes in health facility users’ infant feeding views and practices over time. These should increase participation of AGYW, pregnant women and men, and classify all FGDs according to HIV status and age groups.

## Disclaimer

The findings and conclusions in this report are those of the author(s) and do not necessarily represent the official position of the funding agencies*.*

## Data Availability

The datasets generated during and/or analysed during the current study are not publicly available due confidentiality concerns but are available from the corresponding author on reasonable request.

## Appendix A

**Table 1:** Characteristics of respondents targeted for inclusion in the focus group discussions for PMTCT Option B+ Evaluation (main study) in South Africa 2018

Characteristics	Group 1 Older WHIV (aged > 24 years)	Group 2 Adolescent girls and young women living with HIV AGYWHIV (aged 24 years and younger)	Group 3 Pregnant women regardless of age and HIV status	Group 4 Men/ Male partners regardless of age and HIV status	Group 5 Older WNHIV (aged > 24 years)	Group 6 Adolescent girls and young women not living with HIV (AGYWNHIV) (aged 24 years and younger)
Female	√	√	√		√	√
Male				√		
HIV positive	√	√	√			
HIV negative			√		√	√
Mother pregnant			√			
Mother’s age						
< 20 years		√	√			√
20–24 years,		√	√			√
25–29 years	√		√		√	
30–34 years	√		√		√	
> 35 years	√		√		√	
Mother and baby 0–6 months	√	√			√	√
Father with baby 0–6 months				√		
Lived in district for > 6 months	√	√	√	√	√	√

Adolescent girls and youngwomen- 24 years and below; Adult/older: 25 years and older. All groups (except for the group of pregnant women) should have had babies aged 0–6 months and should have been living in the district for > 6 months

## Appendix B

Option B+ − Qualitative Component FGD topic guide.

- Self- introduction - facilitator and co-facilitator and ask the group members to introduce themselves.
- State the aim of the focus group: Through this discussion we hope to understand your experiences and attitudes towards the test and treat strategy for al HIV positive pregnant and lactating women. Your names will not be documented.
- Go through, the consent process.
- Proceed with those giving consent.

Group 1 - Postnatal Women living with HIV (25 yrs and above).

Section 2: Policy knowledge and experience of care under the B+ policy: This section aims to gather information about their direct experience of PMTCT-related care, in terms of availability of supplies,

- Please share your personal experiences regarding PMTCT services from the time you started ANC in this facility? Probe: access and quality of care (ANC/HIV counselling and testing, viral load monitoring/Infant feeding counselling/ Drug supply and ART adherence counselling/immunisations and EID/family planning services/social support/referral and transfer).

Section 2: Effect of B+ on the health system and community: In your experience and opinion

- What are your perceptions and experiences regarding PMTCT Option B+ effect on mothers, babies, men/fathers and clinics/hospitals? Probe: their perceptions about test and treat, health status, adherence, disclosure, stigma, infant feeding, family planning, pregnancy preparedness?
- How does your community view people who are taking ARVs? (PROBE – stigma, discrimination, logistical issues for reaching clinic, treatment adherence and retention in care)?
- What are the perceptions of healthcare workers for mothers who are taking ARVs Probe: what is the effect of these perceptions for mothers and their infants (stigma, discrimination, logistical issues for reaching clinic, access to care, quality of care, treatment adherence and retention in care)?

Section 3: Sustaining care under B+

- What experiences have you heard or seen from people (mothers/fathers/infants) who are taking ARVS for life? Probe: their beliefs about ARVs, adherence (enablers, successes and challenges), alternative HIV treatments (traditional medicines, spiritual healers), social support.
- With relevance to access to health care, treatment adherence and attending clinic appointments, what positive experiences have you seen or heard of? What negative experiences have you seen or heard of?

If respondents speak freely about their HIV status and being on ART – you may ask the questions in red.

Section 4: Personal experiences of being on ART for life

- Please share your experiences about being on ART for life? Probe: what it means for you, your infant and partner and other immediate family members, what have you experienced since you started taking treatment, how do you ensure that you are taking treatment correctly, where do you collect your treatment and how often how often, how do you manage clinic appointments scheduled during working hours/clinic appointments clutching with other commitments?
- What could enable you and your infant to adhere to treatment and clinic appointments? Probe: what can be improved or changed, what are your support systems?

Section 5: Communication and experiences with mobile technology as a tool to support adherence or promote health care seeking or communicating results.

- To promote access to care, retention and optimum ART adherence, how would you prefer to get PMTCT/ART information, care and treatment and why? Probe: use of mobile technology/internet, IEC material, appointment schedules, social support, clinic operating hours, quality of service.
- What could be the benefits and challenges of receiving health information (like reminders for clinic appointments, collection of results, adherence) using cell-phones? What other sources of information regarding PMTCT/ART can be useful?

Section 6: Closing questions

- Tell us about any other experiences related to accessing maternal and child health care and how these impact on your health?
- What else would you like us to know about PMTCT/ART services?
- Do you have any questions to ask us?

Group 2 – Postnatal Adolescent girls and young women living with HIV (24 yrs and below).

Section 2: Policy knowledge and experience of care under the B+ policy: This section aims to gather information about their direct experience of PMTCT-related care, in terms of availability of supplies,

- Please share your personal experiences regarding PMTCT services from the time you started ANC in this facility? Probe: access and quality of care (ANC/HIV counselling and testing, viral load monitoring/Infant feeding counselling/ Drug supply and ART adherence counselling/immunisations and EID/family planning services/social support/referral and transfer).

Section 2: Effect of B+ on the health system and community: In your experience and opinion

- What are your perceptions and experiences regarding PMTCT Option B+ effect on mothers, babies, men/fathers and clinics/hospitals? Probe: their perceptions about test and treat, health status, adherence, disclosure, stigma, infant feeding behaviours among adolescent girls and young women
- How does your community view people who are taking ARVs? Probe: what is the effect of these perceptions (stigma, discrimination, logistical issues for reaching clinic, treatment adherence and retention in care)?
- What are the perceptions of peers (other adolescent girls and young women) with regards to adolescent girls and young women who are taking ARVs Probe: what is the effect of these perceptions for young mothers and their infants (stigma, discrimination, logistical issues for reaching clinic, treatment adherence and retention in care)?
- What are the perceptions of healthcare workers for adolescent girls and young women who are taking ARVs Probe: what is the effect of these perceptions for young mothers and their infants (stigma, discrimination, logistical issues for reaching clinic, access to care, quality of care, treatment adherence and retention in care)?

Section 3: Sustaining care under B+

- What experiences have you heard or seen from people (mothers/fathers/infants) who are taking ARVS for life? Probe: their beliefs about ARVs, adherence (enablers, successes and challenges), alternative HIV treatments (traditional medicines, spiritual healers), social support.
- With relevance to access to health care, treatment adherence and attending clinic appointments, what positive experiences have you seen or heard of? What negative experiences have you seen or heard of? What have been your personal experiences? What can be improved or changed?

If respondents speak freely about their HIV status and being on ART – you may ask the questions in red.

Section 4: Personal experiences of being on ART for life

- Please share your experiences about being on ART for life? Probe: what it means for you, your infant and partner and other immediate family members, what have you experienced since you started taking treatment, how do you ensure that you are taking treatment correctly, where do you collect your treatment and how often how often, how do you manage clinic appointments scheduled during working hours/clinic appointments clutching with other commitments, what are your support systems?
- What could enable you and your infant to adhere to treatment and clinic appointments? Probe: what can be improved or changed?
- Section 5: Communication and experiences with mobile technology as a tool to support adherence or promote health care seeking or communicating results.
- To promote access to care, retention and optimum ART adherence, how would you prefer to get PMTCT/ART information, care and treatment and why? Probe: use of mobile technology/internet, IEC material, appointment schedules, social support, adolescent targeted interventions, clinic operating hours, quality of service.
- What could be the benefits and challenges of receiving health information (like reminders for clinic appointments, collection of results, adherence) using cellphones? What other sources of information regarding PMTCT/ART can be useful?

Section 6: Closing questions:

- Tell us about any other experiences related to accessing maternal and child health care and how these impact on your health.
- What else would you like us to know about PMTCT/ART services?
- Do you have any questions to ask us?
  Group 3 – Pregnant women includes women living- and not living with HIV.

Section 2: Policy knowledge and experience of care under the B+ policy: This section aims to gather information about their direct experience of PMTCT-related care, in terms of availability of supplies,

- Please share/describe your perceptions regarding PMTCT services/programme from the time you started ANC in this facility.
- How do PMTCT services impact on other clients who are not part of the PMTCT programme in this facility?

Section 3: Effect of B+ on the health system and community: In your experience and opinion

- What are your perceptions and experiences regarding PMTCT Option B+ effect on mothers, babies, men/fathers and clinics/hospitals? Probe: their perceptions about test and treat, health status, adherence, disclosure, stigma, infant feeding.
- How does your community view people who are taking ARVs? Probe: How do these perceptions impact on (stigma, discrimination, logistical issues for reaching clinic, treatment adherence).
- How do health workers perceive people who are taking ARVs? Probe: How do these impact on (stigma, discrimination, accessing care, quality of services, treatment adherence and retention in care)?

Section 4: Sustaining care under B+

- What experiences have you heard or seen from pregnant mothers who are taking ARVS for life? Probe: their beliefs about ARVs, adherence, clinic appointments (enablers, successes and challenges), alternative HIV treatments (traditional medicines, spiritual healers), social support.

Section 5: Closing questions:

- Do you have any questions to ask us?

Group 4 - men / male partners with children less than 6 months.

Section 2: Policy knowledge and experience of care under the B+ policy: This section aims to gather information about their direct experience of PMTCT-related care, in terms of availability of supplies,

- Please share your personal experiences regarding PMTCT services for pregnant and post-partum mothers and their infants? Probe: access and quality of care (ANC/HIV counselling and testing, viral load monitoring/Infant feeding counselling/ Drug supply and ART adherence counselling/immunisations and EID/family planning services/social support/referral and transfer, male partner testing).

Section 2: Effect of B+ on the health system and community: In your experience and opinion

- What are your perceptions and experiences regarding PMTCT Option B+ effect on mothers, babies, men/fathers and clinics/hospitals? Probe: their perceptions about test and treat, health status, treatment adherence, disclosure, stigma, infant feeding, family planning, pregnancy preparedness?
- How does your community view people who are taking ARVs? (PROBE – stigma, discrimination, logistical issues for reaching clinic, treatment adherence and retention in care)?
- What are the perceptions of healthcare workers for mothers who are taking ARVs Probe: what is the effect of these perceptions for mothers and their infants (stigma, discrimination, logistical issues for reaching clinic, access to care, quality of care, treatment adherence and retention in care)?

Section 3: Sustaining care under B+

- What experiences have you heard or seen from people (mothers/fathers/infants) who are taking ARVS for life? Probe: their beliefs about ARVs, adherence (enablers, successes and challenges), alternative HIV treatments (traditional medicines, spiritual healers), social support.
- With relevance to access to health care, treatment adherence and attending clinic appointments, what positive experiences have you seen or heard of? What negative experiences have you seen or heard of?

If respondents speak freely about their HIV status and being on ART – you may ask the questions in red.

Section 4: Personal experiences of being on ART for life

- Please share your experiences about being on ART for life? Probe: what it means for you, your infant and partner and other immediate family members, what have you experienced since you started taking treatment, how do you ensure that you are taking treatment correctly, where do you collect your treatment and how often how often, how do you manage clinic appointments scheduled during working hours/clinic appointments clutching with other commitments?
- What could enable you, your partner and your infant to adhere to treatment and clinic appointments? Probe: what can be improved or changed, what are your support systems?

Section 5: Communication and experiences with mobile technology as a tool to support adherence or promote health care seeking or communicating results.

- To promote access to care, retention and optimum ART adherence, how would you prefer you, your partner to get PMTCT/ART information, care and treatment and why? Probe: use of mobile technology/internet, IEC material, appointment schedules, social support, clinic operating hours, quality of service.
- To promote family orientated care, treatment adherence and retention in care for mothers, infants and their partners, what could be a role male partners? What can be introduced, improved or changed?
- What could be the benefits and challenges of receiving health information (like reminders for clinic appointments, collection of results, adherence) using cell-phones? What other sources of information regarding PMTCT/ART can be useful?

Section 6: Closing questions:

- Tell us about any other experiences related to accessing care and ARVs? how these impact on your health?
- What else would you like us to know about PMTCT/ART services?
- Do you have any questions to ask us?

Group 5 - Postnatal women not living with HIV (25 yrs and above).

Section 2: Policy knowledge and experience of care under the B+ policy: This section aims to gather information about their direct experience of PMTCT-related care, in terms of availability of supplies,

- Please share/describe your perceptions regarding PMTCT services/programme from the time you started ANC in this facility.
- How do PMTCT services impact other clients who are not part of the PMTCT programme in this facility?

Section 3: Effect of B+ on the health system and community: In your experience and opinion

- What are your perceptions and experiences regarding PMTCT Option B+ effect on mothers, babies, men/fathers and clinics/hospitals? Probe: their perceptions about test and treat, health status, adherence, disclosure, stigma, infant feeding.
- How does your community view people who are taking ARVs? Probe: How do these perceptions impact (stigma, discrimination, logistical issues for reaching clinic, treatment adherence).
- How do health workers perceive people who are taking ARVs? Probe: How do these impact on (stigma, discrimination, accessing care, quality of services, treatment adherence and retention in care)?

Section 3: Sustaining care under B+

- What experiences have you heard or seen from people (mothers/fathers/infants) who are taking ARVS for life? Probe: their beliefs about ARVs, adherence (enablers, successes and challenges), alternative HIV treatments (traditional medicines, spiritual healers), social support.
- With relevance to treatment adherence and attending clinic appointments, what positive experiences have you seen or heard of? What negative experiences have you seen or heard of?

Section 5: Closing questions:

- Do you have any questions to ask us?

Group 6 - Postnatal adolescent girls and young women not living with HIV (24 yrs and below).

Section 2: Policy knowledge and experience of care under the B+ policy: This section aims to gather information about their direct experience of PMTCT-related care, in terms of availability of supplies,

- Please share/describe your perceptions regarding PMTCT services/programme from the time you started ANC in this facility.
- How do PMTCT services impact other clients who are not part of the PMTCT programme in this facility?

Section 3: Effect of B+ on the health system and community: In your experience and opinion

- What are your perceptions and experiences regarding PMTCT Option B+ effect on mothers, babies, men/fathers and clinics/hospitals? Probe: their perceptions about test and treat, health status, adherence, disclosure, stigma, infant feeding?
- How does your community view people who are taking ARVs? Probe: How do these perceptions impact on (stigma, discrimination, logistical issues for reaching clinic, treatment adherence)?
- What are the perceptions of peers (other adolescent girls and young women) with regards to adolescent girls and young women who are taking ARVs Probe: what is the effect of these perceptions for young mothers and their infants (stigma, discrimination, logistical issues for reaching clinic, treatment adherence and retention in care)?
- How do health workers perceive people who are taking ARVs? Probe: How do these impact on (stigma, discrimination, accessing care, quality of services, treatment adherence and retention in care)?

Section 3: Sustaining care under B+

- What experiences have you heard or seen from young mothers, fathers and infants who are taking ARVS for life? Probe: their beliefs about ARVs, adherence (enablers, successes and challenges), alternative HIV treatments (traditional medicines, spiritual healers), social support.
- With relevance to treatment adherence and attending clinic appointments, what positive experiences have you seen or heard of? What negative experiences have you seen or heard of?

Section 5: Closing questions:

- Do you have any questions to ask us?

## Appendix C

**Table 2:** Number of  FGDs per category of participants conducted and total number of participants in each category of FGDs

Districts	Group 1: 8–10 Women living with HIV and are 25 years old and above with babies aged 0–6 months old who use health services in the district and have lived in this district for more than 6 months = OLDER Postnatal WHIV	Group 2: 8–10 Women living with HIV who are 24 years old or less with babies aged 0–6 months old who use health services in district and have lived in this district for more than 6 months Postnatal AGYWHIV	Group 3: 8–10 pregnant women (HIV positive and negative) who use health services in district and have lived in this district for more than 6 months, PREGNANT WOMEN regardless of age and HIV status	Group 4: 8–10 men with children younger than 6 months who use health services in district and have lived in this district for more than 6 months MEN/male partners. Regardless of age and HIV status	Group 5: 8–10 HIV negative mothers (with documented HIV negative test result) who are 25 years old and above with babies aged 0–6 months old who use health services in district and have lived in this district for more than 6 month Postnatal OLDER WNHIV	Group 6: 8–10 HIV negative mothers (with documented HIV negative test) who are 18–24 years old with babies aged 0–6 months old who use health services in district and have lived in this district for more than 6 months Postnatal AGYWNHIV
Bojanala (North- West)			1		1	
Ehlanzeni (Mpumalanga)	1**		1	1**	1	
Ekhuruleni (Gauteng)	1		1		1	
Ethekwini (KwaZulu-Natal)	1 (Incl 2 AGYWHV)**		1	1**	1	1
Greater Sekhukhune (Limpopo)	1		1			
ORTambo (Eastern Cape)	1		1		1	
TOTAL number of FGDS	5	0	6	2	5	1
Total number of participants in each category interviewed	28 (incl 2 AGYWHIV)	0	37	9	34	5

Adolescent girls and young women- 24 years and below; Adult/older: 25 years and older. All groups (except for the group of pregnant women) should have had babies aged 0–6 months and should have been living in the district for > 6 months

**FGDs consisted of between less than 6 respondents the two for men, one for older postnatal WHIV and one combined postnatal group of AGYWNHIV and AGYWHIV

## Appendix D

**Table 3:** Infant feeding themes and sub-themes

Main Themes	Sub-themes
3.1.1 Knowledge and practices about the recommended breastfeeding and breastfeeding duration	
3.1.2 Exclusive breastfeeding knowledge and practices	
3.1.3Breastfeeding and mother-to-child transmission of HIV (MTCT)	
	*3.1.3.1 Fear of infecting the baby*
	*3.1.3.2 Effectiveness of ARVs to prevent HIV transmission through breastfeeding*
	*3.1.3.3 Challenges with ART adherence*
	*3.1.3.4 Poor viral load monitoring*
	*3.1.3.5 HIV status disclosure*
3.1.4 Perceived quality of counselling	
	*3.1.4.1 Health care worker counselling*
	*3.1.4.2 Accuracy of infant feeding messages*
	3.1.4.3 Experiences with Mom Connect
3.1.5 Lack of infant feeding / breastfeeding support	
	*3.1.5.1 Support by health care workers*
	*3.1.5.2 The role of male partners*
3.1.6 Other breastfeeding- related challenges impacting on HIV transmission through breastfeeding	
	*3.1.6.1 Unknown partner HIV status*
	*3.1.6.2 Inconsistent or non-condom use*
	*3.1.6.3 Poor HIV risk perception*
3.1.7 Other factors contributing to inappropriate infant feeding practices	
	*3.1.7.1 Perceived insufficient milk and infant cues*
	*3.1.7.2 Schooling and Employment*
	*3.1.7.3 Family pressures*
	*3.1.7.4 Maternal attitudes to breastfeeding*
3.1.8 Coping strategies for mothers	

## Appendix E

**Table 4:** A guide to quantify respondents’ responses

Few	Less than 10% of participants
Several	Less than 20%
Some	More than 20%
Many	Nearly 50%
A majority	More than 50%, but fewer than 75%
Most	More than 75%
Vast majority	Nearly all participants, with some still having different views
Unanimous, or almost all	All participants, or the vast majority gave similar answers and the rest did not comment

Source:: https://www.evalacademy.com/articles/how-to-quantify-qualitative-data

## Abbreviations

AGYWHIV: adolescent girls and young women living with HIV
AGYWNHIV: adolescent girls and young women not living with HIV
ANC: antenatal care
ART: antiretroviral therapy
ARVs: antiretroviral drugs
CDC: Centers for Disease Control and Prevention
EBF: exclusive breastfeeding
FGD: focus group discussions
HCW: health care worker
LMICs: low- and middle-income countries
PMTCT: Prevention of mother-to-child transmission of HIV
SADHS: South African Demographic and Health Survey
SAMRC: South African Medical Research Council
SA NDoH: South African National Department of Health
SSA: Sub-Saharan Africa
UNAIDS: Joint United Nations Programme on HIV/AIDS
WHIV: women living with HIV
WNHIV: women not living with HIV
WHO: World Health Organization.
